# A Short Report on a Single-center Survey of Barium Acute Appendicitis

**DOI:** 10.2188/jea.JE20230334

**Published:** 2024-11-05

**Authors:** Taku Harada, Takashi Watari

**Affiliations:** 1 Division of General Medicine, Nerima Hikarigaoka Hospital, Tokyo, Japan; 2 Division of Diagnostic and Generalist Medicine, Dokkyo Medical University Hospital, Tochigi, Japan; 3 General Medicine Center, Shimane University Hospital, Shimane, Japan; 4 Department of Medicine, University of Michigan Medical School, Ann Arbor, Michigan, USA

Japan has a higher mortality of gastric cancer than Western countries.^1^ As of 2019, gastric cancer was ranked as the second and fourth leading cause of cancer-related death among males and females in Japan, respectively.^2^ Since 2014, upper gastrointestinal endoscopy (GIE) has been recommended as a screening test for gastric cancer to replace barium examination.^3^ However, as of 2016, barium examination has been performed ∼10 times more frequently than GIE in Japan and continues to play a leading role in gastric cancer screening.^4^ Barium examination was associated with a 1.5-fold increased risk of acute appendicitis within the first year after screening, with a specific 10-fold higher risk in the first 2 months after screening.^5^

**Figure 1.**
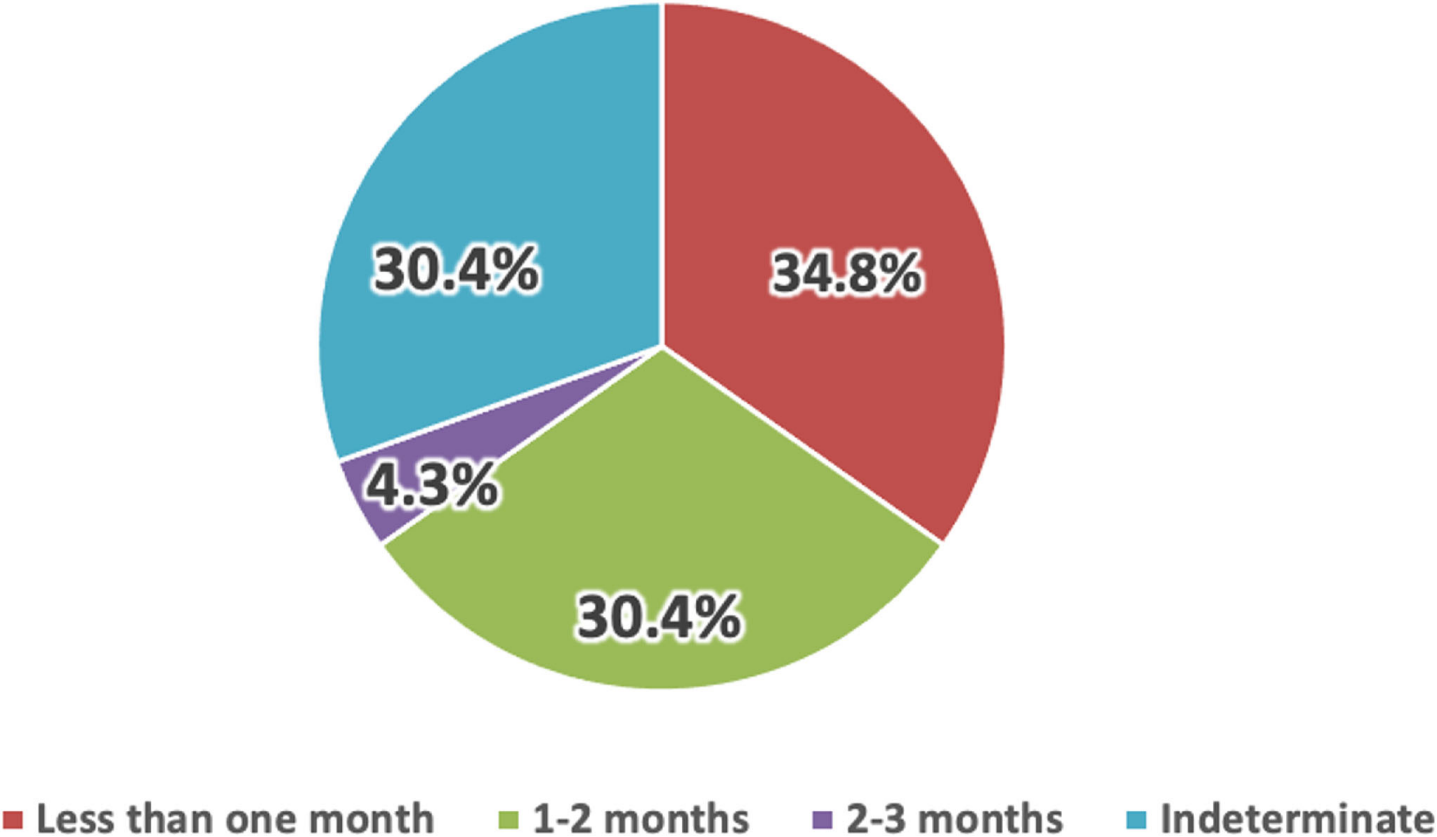
Time delay from barium ingestion to appendicitis onset

Acute appendicitis is the most common cause of acute abdomen in adults.^6^ Katagiri et al^7^ have reported that acute barium appendicitis occurs in 3% of appendicitis cases. In all cases, high-absorption material (>3,000 Hounsfield units on computed tomography [CT]) was identified within the appendix. Barium examination, frequently employed for gastric cancer screening, was associated with an incidence (3%) of acute appendicitis; however, the study was conducted at a single institution, and the prevalence needs to be confirmed at other institutions in Japan. Herein, we investigated the epidemiology of acute barium appendicitis at an urban hospital in Japan. We retrospectively analyzed previously reported^8^ observational data on causes of adult acute appendicitis treated at an acute care hospital from April 1, 2014, to March 31, 2021. Barium appendicitis was defined according to the criteria established by Katagiri et al^7^: presenting with acute appendicitis symptoms, history of barium examination, and confirmation of high-absorption material within the appendix on CT imaging. This study was approved by the ethical review board of the Showa University Koto Toyosu Hospital and conducted according to the Declaration of Helsinki. Ethical considerations included obtaining informed consent using an opt-out method. Among the 763 adult patients diagnosed with acute appendicitis, barium appendicitis was confirmed in 23 (3%). Table 1 presents clinicopathological characteristics of the 23 identified patients. Of the 23 patients, 6 (26.1%) were female. The median patient age was 40 (interquartile range, 37–50) years. Although barium examination for gastric cancer screening is recommended for individuals ≥40 years in Japan, eight patients (34.8%) were aged <40 years. The time between barium examination and the onset of acute appendicitis was known in 16 patients: <1 month in 8 patients (34.8%) and between 1–2 months in 7 patients (30.4%; Figure 1). Among the 23 patients, one had undergone previous conservative treatment for appendicitis.

**Table 1.** Characteristics of patients with barium appendicitis

Age	Sex	Time delay from barium ingestion to appendicitis onset	Pathology	Hospitalization
61	Female	Slightly more than a month	Gangrenous appendicitis	+
54	Male	Indeterminate	—	+
42	Male	One and a half months.	Phlegmonous appendicitis	+
43	Male	26 days	Gangrenous appendicitis	+
50	Male	16 days	Gangrenous appendicitis	+
46	Male	Indeterminate	—	−
35	Male	Slightly more than two months	Gangrenous appendicitis	+
45	Male	40 days	—	+
43	Male	Indeterminate	Gangrenous appendicitis	+
40	Male	18 days	Phlegmonous appendicitis	+
54	Female	Indeterminate	—	−
37	Female	36 days	—	+
55	Male	One week	Phlegmonous appendicitis	+
43	Male	3 weeks	Gangrenous appendicitis	+
35	Male	Indeterminate	—	−
31	Male	Indeterminate	—	+
65	Male	3 days	Phlegmonous appendicitis	+
39	Male	About 3 weeks	Gangrenous appendicitis	+
48	Female	3 days	—	−
40	Female	Indeterminate	—	+
27	Male	32 days	Phlegmonous appendicitis	+
33	Male	31 days	Phlegmonous appendicitis	+
37	Female	Within a month	Gangrenous appendicitis	+

Importantly, our study identified that barium appendicitis accounts for 3% of all cases of adult acute appendicitis, consistent with previous reports.^7^ In Japan, although both upper GIE and barium examination are used for gastric cancer screening, the risk of appendicitis is not widely recognized as an adverse event of barium ingestion.^3^^,^^9^ Although gastric cancer screening is indicated for individuals ≥40 years,^3^ our study included eight patients with acute barium appendicitis aged <40 years. In these cases, barium examination was not preventive and likely self-financed. The rationale for performing barium screening for gastric cancer needs to be considered against the risk for acute barium appendicitis among individuals <40 years of age, given the low probability of gastric cancer in these individuals.

Our study has three limitations. First, as a retrospective observational study based on existing records, our analysis risks data gaps and reduced reliability. Second, similar to Katagiri et al,^7^ the data were obtained from an urban hospital in Japan, which prevents generalization to other regions. Finally, estimating the incidence of barium appendicitis is challenging after barium testing, due to the characteristics of this research.
